# Fetal Movement Counting Using Optical Fibre Sensors

**DOI:** 10.3390/s21010048

**Published:** 2020-12-24

**Authors:** Chalani L. Abeywardena, Frederique J. Vanheusden, Kate F. Walker, Richard Arm, Qimei Zhang

**Affiliations:** 1 Department of Engineering, School of Science and Technology, Nottingham Trent University, Nottingham NG11 8PR, UK; Chalani.Abeywardena@nottingham.ac.uk (C.L.A.); frederique.vanheusden@ntu.ac.uk (F.J.V.); 2 Optics and Photonics Group, Faculty of Engineering, University of Nottingham, Nottingham NG7 2RD, UK; 3 Department of Obstetrics and Gynecology, University of Nottingham, Nottingham NG7 2UH, UK; Kate.Walker@nottingham.ac.uk; 4 School of Art and Design, Nottingham Trent University, Nottingham NG11 8PR, UK; richard.arm@ntu.ac.uk

**Keywords:** fibre Bragg grating, optical fibre sensing, fetal movement monitoring, kick counting

## Abstract

Daily fetal movement counting based on maternal perception is widely deployed to monitor fetal wellbeing. However, the counting performed by the mother is prone to errors for various reasons. There are limited devices on the market that can provide reliable and automatic counting. This paper presents a prototype of a novel fetal movement monitoring device based on fibre Bragg grating sensors. Deformation of the skin caused by a fetal movement can lead to a change of the strain and stress on the optical fibre sensors, therefore can induce distortions to the breathing pattern of the mother. In the study data was gathered by the sensors through strain measurement and was post-processed using independent component analysis (ICA) and high-pass filtering to show the instances of the fetal movements. Information gathered during user trials with the prototype suggests that the system detects significantly higher numbers of fetus movements than that observed based on the mother’s perception. Among the various techniques available for fetal movement monitoring, fibre optic sensing provides many advantages including multiplex capability, flexibility and minimal size, making the concept an attractive solution for reliable monitoring of antenatal fetal movements.

## 1. Introduction

Stillbirth rates are a robust indicator of the quality of the provision of maternal health care of any country. According to the 2015 World Health Organization estimates, 2.6 million babies are stillborn across the world annually [1]. In the UK, the stillbirth rate was 4.0 stillbirths per 1000 births in 2018 [2]. A major problem faced by obstetricians is the identification of women who are at risk. Maternal perception of fetal movement is a widely deployed marker of fetal well-being. In one study investigating factors associated with intrauterine fatal deaths, 55% of stillbirth cases occurred in women who presented with a reduction or absence of fetal movement [3]. In another case-control study, it was found that women who reported increased strength of movements in the last 2 weeks had decreased risk of late stillbirth compared with those whose movements were unchanged [4]. Women who perceived their fetus to be quiet in the evening have an almost four-fold increased odds of late stillbirth [5]. Reduction of fetal movement also correlates with fetal hypoxia, fetal growth restriction, umbilical cord complications, being small for gestational age and long-term neurodevelopmental impairment [5,6,7,8,9]. In addition to reduction of fetus movements, there is also a hypothesis that a sudden episode of excessive fetal activity indicates fetal compromise, for example, representing fetal seizures, which if it persists can lead to fetal death [10].

Saastad et al. discuss the significance of counting fetal movements to prevent fetal growth restriction and adverse perinatal outcomes [7]. An increased identification of fetal growth restriction and improved perinatal outcomes, without inducing consultations or obstetric interventions were reported [7]. The World Health Organization also recommends clinical enquiry by antenatal care (ANC) providers of maternal perception of fetal movement during the ANC visit and healthy pregnant women reporting reduced fetal movements [11].

Fetal movement begins at about seven to eight weeks of gestation [12,13]. These early movements occur at one or two poles of the fetus as small, slow displacements. These movements will disappear until about 15 gestational weeks, where more robust patterns of fetal movement start to appear. Some examples of fetal movement include general body movements, startle and twitch movements, isolated limb movements, breathing movements, hiccups, isolated head and neck movements, hand-face contact, stretch and rotation, etc. [14]. Three major physical, environmental influences have been shown to affect fetal movement. The amount of free intrauterine space is dependent on the uterine and fetal size, the amount and location of the amniotic fluid and fetal positioning [13]. Moreover, external effects including maternal nutrition consumption and acoustic stimulation can also influence fetal movements [13].

Despite these variable influences on the perception of movement, experiential maternal perception of daily fetal movement remains a significant tool for antenatal fetal wellbeing monitoring [15,16]. But it was found that the maternal perception has great variations that are between 2.4% and 81.0% (median 44.8%) of movements observed on ultrasound scan [17]. In addition to the physical and environmental factors, maternal factors such as maternal employment can also influence an individual’s interpretation of sensation because busy mothers tend not concentrate on fetal activity [18]. Time of perception is another influencing factor as most mothers reported perceiving weak fetal movements at night, and increased movements in the early morning [18]. In the latter case mothers are likely to be in bed and external distractions are likely to be minimized.

There are only a handful of commercial, professionally recognized products available for identification and quantification of fetal movements. Ultrasound technology is the most widely used technique for fetal movement monitoring. It has been shown that there is a significantly positive correlation between the number of movements recorded by ultrasound equipment, compared to the recorded subjective interpretation of fetal movement felt by the mother [19]. Advancements in fetal magnetic resonance imaging (MRI) through cine-MRI scans allow direct monitoring of the movements of the entire fetus [20]. However, both techniques are expensive and have limited accessibility. Moreover, these methods are not suitable for continuous and prolonged monitoring due to their large size, complexity and accumulative exposure health risks [21]. Therefore, these technologies are mostly used to monitor fetal movement in clinical settings and are only performed by skilled practitioners.

Borges et al. [22] report on two wearable sensing technologies for fetal movement monitoring in low-risk pregnancies: one with flex sensors and the other with piezoelectric sensors. However, it was found that some fetal movements perceived by mothers were not detected by the sensors. And weak fetal movements were easily hidden by signals with larger amplitude such as those due to mother’s activities. Ryo et al. developed capacitive acceleration-based sensors which have two electrodes, one is a fixed backplate and the other is a moveable diaphragm that was used to detect the oscillations of the abdomen wall [23]. High agreement was claimed for gross fetal movements of the trunk between the sensors and ultrasonography. However, the sensors are slightly bulky with a weight of 20 g, dimensions of 2.8 cm, and an obvious thickness. Lai et al. [24] discussed the performance of a wearable acoustic system for fetal movement discrimination. The technique is based on measuring vibrations resulting from fetal movements using eight acoustic sensors and a tri-axial accelerometer to remove the mother’s activity. The system was found to be sensitive in detecting fetal startle movements (quick generalized movement, lasting about a second), but not sensitive enough to detect whole-body movements. Furthermore, these results were not compared to the mother’s sensorial interpretation of recorded movements.

One technology that may help reliably monitor fetal movements is the application of fibre-optic sensing. Optical fibres adopted as flexible and miniature sensing elements have been used successfully in other medical device applications [25]. The field of fibre-optic technology has undergone remarkable growth over the last couple of decades, particularly in healthcare applications, and their use has been shown to offer many advantages over competing technologies. Some of these advantages are biocompatibility, small size, flexibility, no electromagnetic interference, and multiplex capability [26].

The objective of this work is to investigate the possibility of a novel fetal movement monitoring belt using fibre optic technology based on fibre Bragg grating (FBG) sensors. The results from the fibre-optic sensors were compared with the mother’s perception. FBGs are periodic variations of the refractive index in the core of the optical fiber. When the broadband light is launched into the fibre core, only a narrow bandwidth is reflected. The relationship between the centre wavelength of the reflected light (i.e., Bragg wavelength) from an FBG, the effective refractive index (n) and the grating period (Λ, the periodicity of the index modulation) can be given by using the Bragg equation [27]:

λ_B_ = 2nΛ.

When the FBG is strained or stressed it will cause a change in the period of the FBG, and hence a change in the Bragg wavelength. In the current study, this sensing phenomena has been utilized to detect fetal movements.

## 2. Materials and Methods

### 2.1. Design of the Fibre-Optic Based Sensing Structure

The prototype fibre-optic sensor array used in this study consists of eight FBG sensors which had center wavelengths of 1518 nm, 1527 nm, 1536 nm, 1545 nm, 1554 nm, 1562 nm, 1572 nm, 1581 nm respectively. The FBGs are femtosecond-written in a bend insensitive optical fibre with polyimide coating (SM1250BI, Fibercore, Southampton, UK). Polyimide coating was chosen as such coating will not exhibit any strain loss due to slippage between the coating and cladding [28]. All the FBGs have a length of 3 mm and the reflectivity above 40%.

**Figure 1:**
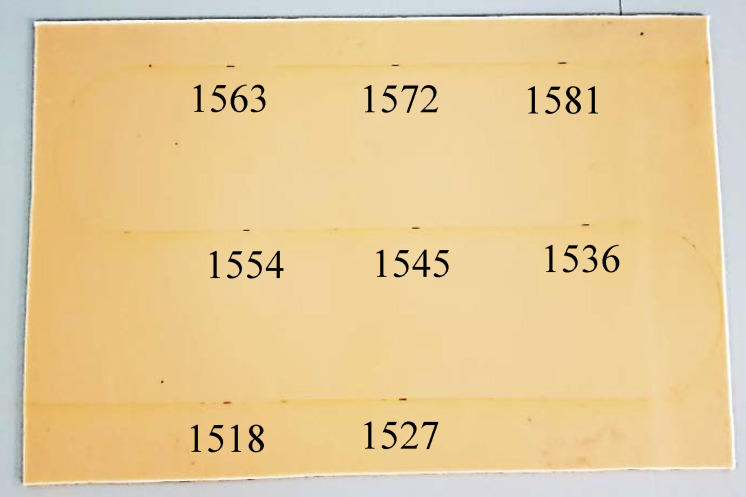
Fibre optic sensor with eight FBGs secured on the membrane. The center wavelength (in nm) of each sensor is shown.

The sensor array was secured to a self-adhesive polydimethylsiloxane (PDMS) gel membrane using a Scotch magic tape (3M, Saint Paul, MN, USA) as illustrated in Figure 1.

The membrane substrate was made from a skin-safe PDMS gel (Platsil^®^ gel 10, Polytek^®^ Development Corp., Easton, PA, USA) prepared according to the methods reported by Arm et al. [29] using commercially available liquid and fibre additives. The same methods and materials were used to create the composite elastomer membranes for these experiments because they have been shown to mimic the Young’ s modulus of human skin [29].

The change in the initial grating period of the FBG is inversely proportional to the Young’s modulus of the supporting structure, as suggested by Equation (4) in [30]. Therefore, to get the optimized sensitivity to the fetal movement, it is preferred to use a supporting structure with a relatively small Young’s modulus. The membrane used in this experiment exhibits an elastic Young’s modulus of 0.22 MPa (±0.1 MPa), where the literature reports that human skin has a wide-ranging modulus across the body, ranging between 0.014 MPa and 0.6 MPa [31,32].

The self-adhesive nature of the composite membrane allowed for a safe, secure, but temporary bond to each volunteers’ skin while gathering data. The soft, elastic modulus of the membrane also ensured unencumbered soft-tissue mobility in all volunteers throughout the experiment, whilst ensuring the sensors remained in place.

### 2.2. Sensitivity Test

**Figure 2:**
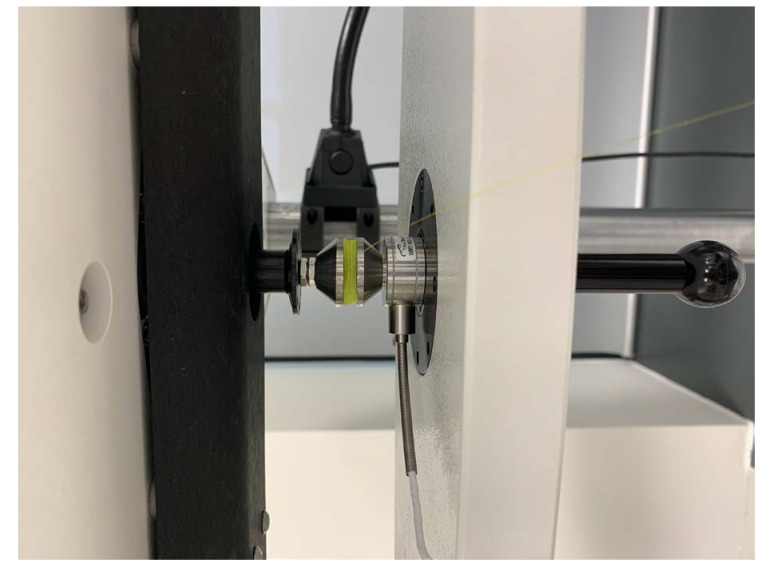
Compression test of the FBG secured on the PDMS gel membrane using a mechanical tester.

During a fetal movement instance, the FBG secured membrane experiences stress both along the longitudinal direction and in the perpendicular direction to the optical fibre. The strain sensitivity of the FBG along optical fibre is about 1.2 pm/µε as suggested by Surre et al. [33]. The sensitivity of the FBGs secured on the PDMS gel membrane to the force applied perpendicular to the membrane was tested using a mechanical test instrument (ElectroForce 3200, TA Instruments, New Castle, DE, USA). A compression force was applied using a compression plate to a membrane patch of 22 mm (length) × 22 mm (width) × 4 mm (thickness), as shown in Figure 2. A ramp force with a rate of 0.1 mm/s and the maximum displacement of 3 mm was applied. The load and the displacement of the mechanical tester were recorded with a sampling frequency of 50 points per second. The Bragg wavelengths of the FBG were recorded by the interrogator simultaneously. The data from the mechanical tester and the interrogator were synchronized using the time labels.

### 2.3. Examinations on Volunteers

A total of seven examinations on three volunteers, all with singleton pregnancies were obtained. The gestational weeks for the three volunteers when performing the tests were 28, 28 and 34 weeks respectively. Six examinations on two non-pregnant women were also performed as a comparison. All volunteers gave their informed consent for inclusion before they participated in the study. The study was conducted in accordance with the Declaration of Helsinki, and the protocol was approved by the Research Ethics Committee at Nottingham Trent University on 30 October 2019 (Project identification code: 609).

**Figure 3:**
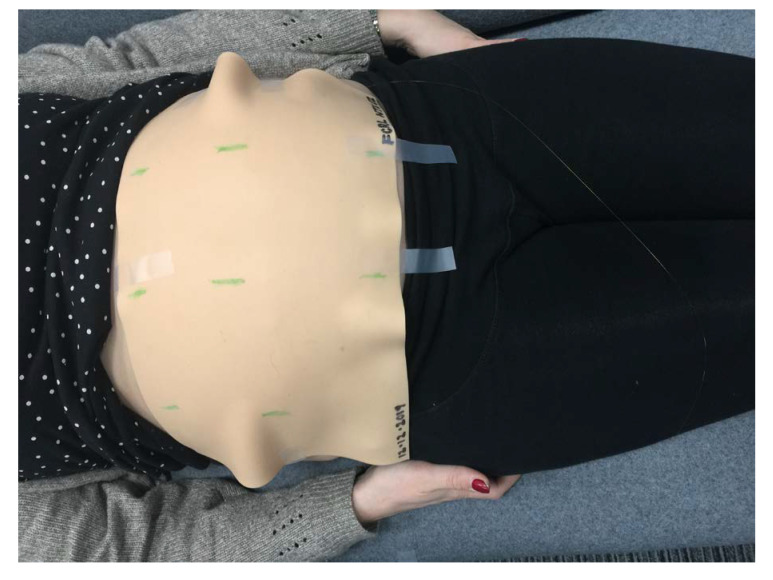
Position of FBG sensor array membrane on volunteer’s abdomen during experiments.

**Figure 4:**
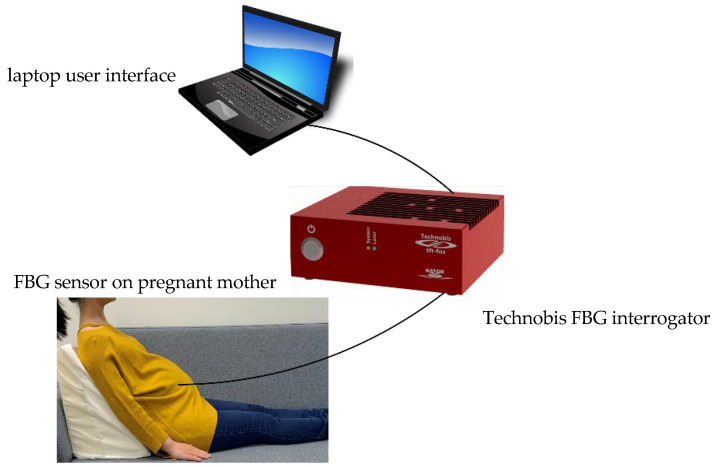
Volunteer experimental setup.

The sensing membrane was gently applied onto the volunteer’s bare abdomen as shown in Figure 3 with the sensors applied to the inner, self-adhesive surface. In this way, the FBG sensors were sandwiched between the membrane and the volunteer’s abdominal skin. The experimental setup is shown in Figure 4.

The FBG sensor was connected to an FBG interrogator (SwitchedGator, Technobis, Alkmaar, The Netherlands). The interrogator was connected to a laptop with a USB connection for data acquisition, logging and visualization and the data was received at 19.23 kHz. Data was logged every 5.2 milliseconds, with the system calculating the average output after every 100 samples.

In order to check recording of the volunteer’s perception, a switch was developed in LabVIEW 2019 (National Instruments, Austin, TX, USA). The volunteer was asked to press the switch by left clicking a mouse each time she felt any fetal movement. The interface of the switch would display a base signal without interaction, and if pressed, it would display a high-level signal. Therefore, the clicks of the volunteer were registered by the system. Switch data was synchronised with the data acquisition from the interrogator.

It has been reported that the greatest number of fetal movements are noted when the mother is lying down [34]. During the test, subjects were placed in a semi-recumbent position to avoid aortocaval compression, as shown in Figure 4. Since the body temperature is much higher than room temperature, the Bragg wavelength signals from the FBGs would increase once applied on the skin. Therefore, before the data recording started, the volunteer was asked to relax and wait for the signals of the FBGs to stabilise. The subjects were asked to lie motionlessly but keep breathing normally. During the measurements if the mother coughed or sneezed, the time this happened would be noted down and the corresponding data would be removed and not counted. No external disturbances were introduced to the measurement system by any means during the test. Each individual test was conducted for ten minutes.

The Bragg wavelength is affected by both the temperature and the strain [27]. When the sensor is attached to the abdomen, it detects lengths changes of the optical fibre and temperature fluctuations in units of wavelength with the interrogator reading the temporal signals of Bragg wavelength. As mentioned earlier, the measurements were started when the detected Bragg wavelength has been stablise indicating the sensor has the same temperature as that of the abdomen skin. In this way the effect of the temperature can be minimised. When the volunteer breathes, a rhythmic wave pattern of wavelength change can be observed. Fetal movement can introduce a distortion by superposing an intensity change over this breathing pattern. Therefore, any distortion on the wavelength pattern is considered as fetal movement.

### 2.4. Post-Processing Algorithms

To identify fetal movement signals effectively, artefacts related to the mother’s breathing should be removed. In this study independent component analysis (ICA) is used to remove the breathing artefacts. In ICA, the sensor signals are separated into components that are statistically independent (or as independent as possible) [35]. As ICA is a form of blind source separation [35], it allows us to extract components without the need for making rigorous assumptions on the original sensor signal.

The principle mathematical description for ICA is that the sensor signals x can be described by a set of independent components multiplied by a weighting matrix A:

x = As

The matrix A can be considered a description of the strength to which each component s1 contributes to a sensor signal x1 at location p1. Independent components can therefore be extracted by calculating the inverse of A (W) and multiplying it with the original sensor signals x:

s = Wx

In the current application, the sensor signals will be separated into independent components, of which some will contain breathing artefact and others will contain fetal movement. It is therefore possible to reconstruct the signal to only contain fetal movement signals by first calculating W, inverting it to A, and setting the weights of A related to breathing signal components to 0. The problem with ICA is that we cannot determine the order of independent components [35]. It is therefore required to manually/visually determine which components should be removed. Another issue is that the variances of the independent components cannot be determined, which can lead to unrealistic (changes in) amplitudes of the reconstructed sensor signals [35].

To extract fetal movement from breathing artefacts, the fast ICA algorithm as described by Hyvarinen was used [36]. The fast ICA algorithm allows a faster convergence in determining the independent components and avoids the need for setting parameters related to the sensor signals’ probability density function [35]. As the sensor device contains eight sensors, eight independent components were estimated and visually inspected for breathing artefact. Weights for components containing artefact were set to 0 and the remaining components were used to reconstruct the fetal movement signal.

To remove the slower trend due to temperature fluctuations a high-pass filter was applied following the ICA processing. According to literatures the breathing rates of the pregnant mothers are 12–21 breaths/min [37], corresponding to a period of 2.86–5 s and a frequency of 0.2–0.35 Hz. The heart rates of pregnant mothers are 60–104 [37] beats/min corresponding to a period of 0.58–1 s and a frequency range of 1–1.73 Hz. The fetal movement duration (time between the beginning and end of each movement) is a few hundreds of milliseconds to a dozen or so seconds, depending on the types of movements [38]. Based on these data the cut-off frequency of the high-pass filter is set to be 2 Hz, to filter the breathing and heart beating signals, while avoid removing fetal movements signals.

The fetal movements can occur anywhere over the abdomen region, and any number of the FBGs can be affected by a fetal movement. Therefore, if there are peaks observed by any FBGs in the ICA data, they are counted as a fetal movement. The output of the sensing system (number of counting) was then compared to the volunteers’ perception.

## 3. Results

**Figure 5:**
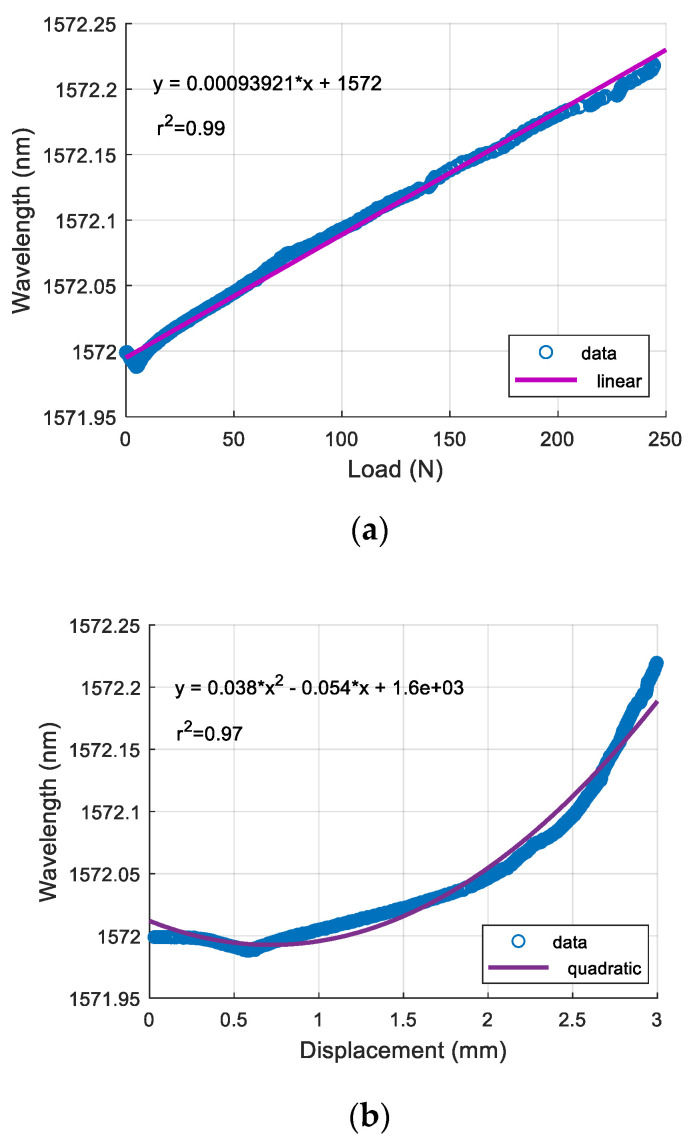
Compression test of the FBG sensor secured on the PDMS gel membrane using the mechanical tester. Linear fitting was used for wavelength vs. load (**a**) with coefficient of determination (R-Squared) of 0.99. Quadratic fitting was used for wavelength vs. displacement (**b**) with R-Squared of 0.97.

The results for the compression tests are shown in Figure 5. The Bragg wavelengths against the loads and the displacements are shown in Figure 5a,b, respectively. It can be seen from Figure 5a that in the range between 4.6 N and 250 N, there is a linear relationship between the Bragg wavelength and the load, and the compression sensitivity is 0.94 pm/N. The slight decrease of the Bragg wavelength of 0.01 nm once the load was applied until it reached 4.6 N is probably due to the bending of the FBG on the soft PDMS structure. A quadratic fitting was applied to express the relationship between the Bragg wavelength and the displacement.

**Figure 6:**
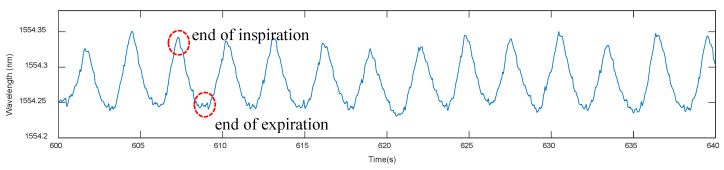
Results from one FBG sensor demonstrating the normal breathing pattern of a pregnant mother while resting. Peaks indicate the end of inspiration, whereas throughs indicate the end of expiration.

**Figure 7:**
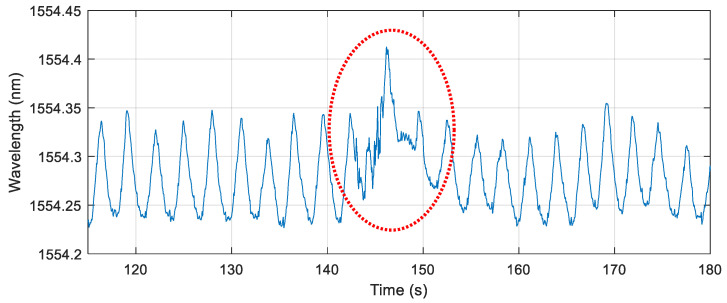
Distortion on the breathing pattern due to a fetal movement.

Figure 6 shows the typical breathing signal observed from one FBG sensor when worn by a pregnant volunteer. A clear rhythmic wave pattern was observed. Peaks identify the end of maternal inspiration, whereas troughs identify the end of breathing expiration, illustrating typical respiratory behavior [39,40]. With the subject in rest and the measurement uninterrupted by external/internal triggers, a very rhythmic breathing signal was observed. A fetal movement inside the uterus can make the pattern became distorted. Exemplary distortions on the breathing pattern are shown in Figure 7.

**Figure 8:**
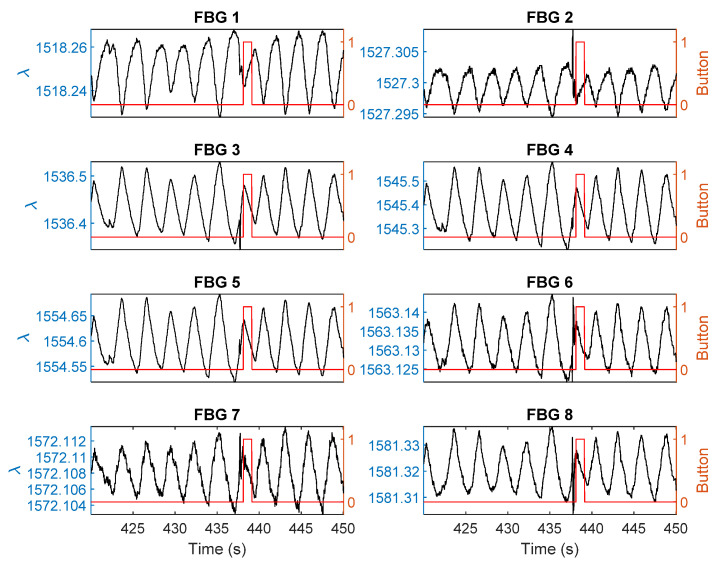
Experimental results for the eight FBG sensors for 30 s showing the breathing signals are distorted due to a fetal movement at 438 s. The black line with the left y axis shows the response of the sensor. The red line with the right y axis shows the mother’s perception.

**Figure 9:**
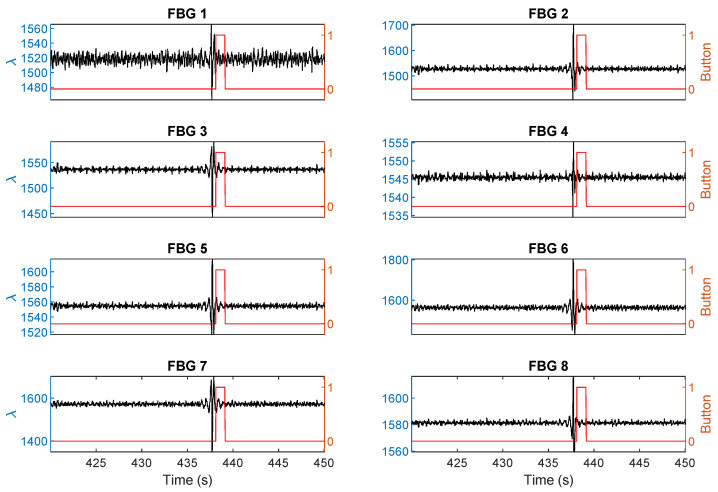
Independent component analysis of the raw sensing signals shown in Figure 8. The black line with the left y axis shows the response of the sensor. The red line with the right y axis shows the mother’s perception.

Figure 8 shows exemplary distortions induced by the fetus at 438 s over all eight sensors during a 30 s measurement window on a pregnant volunteer. During the fetal movement, the breathing signal was interrupted by a distortion in the wavelength causing a sharp peak influenced by the movement. Corresponding ICA data with the breathing signals removed is shown in Figure 9. A clear signal at 437–438 s can be observed indicating a fetus movement. The time when the fetus movement was detected correlates well with mother’s perception, which is indicated by the right y axis.

**Figure 10:**
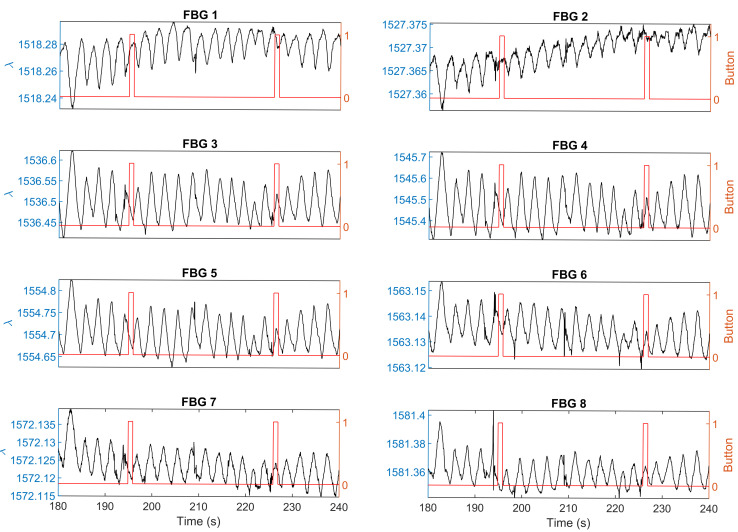
Experimental results show that the mother perceived two movement, while FBG sensors detected three distortions. The black line with the left y axis shows the response of the sensor. The red line with the right y axis shows the mother’s perception.

**Figure 11:**
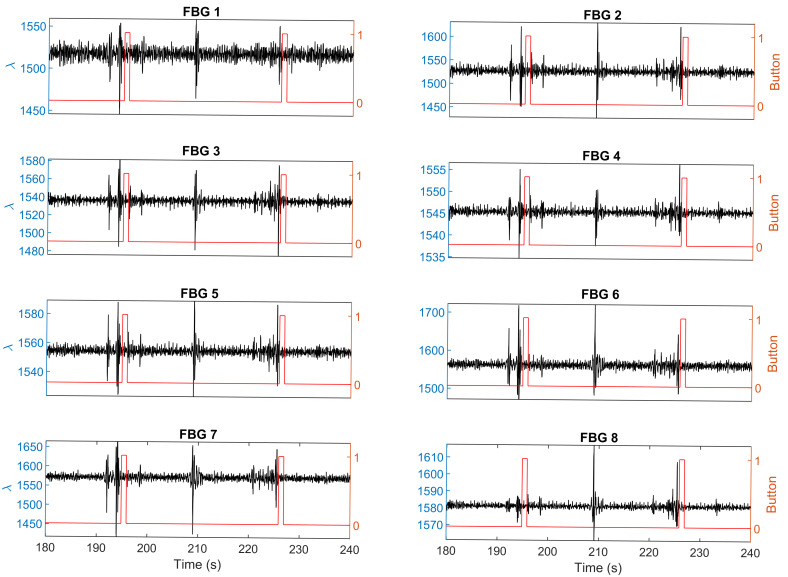
Independent component analysis of the raw sensing signals shown in Figure 10. The black line with the left y axis shows the response of the sensor. The red line with the right y axis shows the mother’s perception. Mother perceived two movements, while FBG sensors detected three movements.

It was found that some fetal movements indicated by distortions of the breathing signal or peaks in the ICA data were missed by the mother. One example has been shown in Figure 10 (raw data) and Figure 11 (ICA data). During this 60-s test, there were two movements recorded by both the sensing system and the mother, which were at 191–199 s and 220–225 s respectively. However, all the eight FBG sensors detected another movement at 208–211 s, which was missed by the mother.

**Figure 12:**
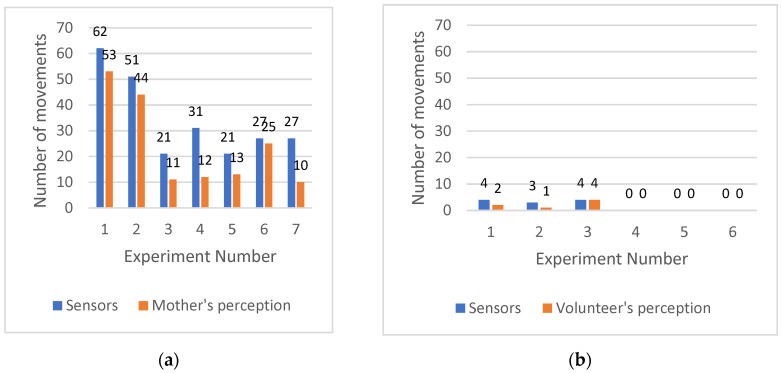
Comparison of number of counts felt by the volunteer and those sensed by the sensors. (**a**) Examinations on pregnant mothers. (**b**) Examinations on non-pregnant women.

Figure 12 compares the number of movements counted using the ICA processed signals and by the volunteers during the 10-min test. Figure 12a,b are examinations on pregnant mothers and non-pregnant women, respectively.

## 4. Discussion

A fetal movement could be identified using the raw data from the sensing structure by observing a distortion to the respiratory signal rhythm. The distortion can be a high intensity sharp peak or a distortion over a long period with a lower intensity, depending on factors such as the types of the movement or gestational age. However, the magnitudes and durations of breathing can change which makes it difficult to identify all the distortions. Heart beating signals of the volunteers can also affect the decision. ICA and high-pass filtering have been used in the study to remove the breathing and heart beating pattern. Clear spike clusters corresponding to distortions to the breathing pattern have been observed indicating fetal movements activities. It is possible to estimate the length and strength of an individual fetal movement from the clusters. For example, the movement shown in the Figure 9 is about 1.7 s. This is within the range of fetal movement duration suggested in literature [38].

As can be seen in Figure 12, in all the experiments on pregnant mothers, the number of counts measured by the optical fibre sensors are higher than that counted based only on the mother’s perception. To determine whether the differences of the two measurements are significant a paired *t*-test was calculated based on Figure 12a which generated a *t*-value of 5.34 and a *p*-value of 0.002. The methods for calculation of *t*-value and *p*-value can be found in literatures [41,42]. Since the *p*-value which indicates the probability of getting a *t*-value of 5.34 by chance is very small (*p*-value < 0.05), it can be concluded that the measurements obtained by the optical fibre sensors are significantly higher than those counted by the mothers. The missed counts as indicated in Figure 10 and Figure 12 may have been low intensity movements such as sucking and swallowing that the mother could barely perceive as movements. It is reasonable to assume also, that human error or loss of concentration could also be attributed to the missing counts in mother’s perception of the movement [43], but the data gathered during these experiments does suggest that the optical fibre sensor was more sensitive and reliable than the mothers’ perception.

In the control examinations on the volunteers that were not pregnant, the number of counts were much less than those produced on pregnant mothers. This suggests that by using the examination protocols described in the Section 2.3, the sensing system picks up only very litter other movements (e.g., volunteer’s body movements) as fetal movements. There were up to 4 counts felt by the volunteer and the sensor, which are most probably due to digestion. The FBG sensors detect the abdominal wall movements or deformation of the abdominal skin. Digestion or abdominal distension due to bloating or trapped wind could cause abdominal wall deformation and therefore interfere with the fetal movement signaling.

It is worth to mention that bowel movement felt by the pregnant mothers could be confused as fetal movements, therefore one may argue that the counts felt by the mothers may not be entirely fetal movements. However, there are only an average of 8.6 bowel movements for women per week [44]. Further, pregnant women often have delayed gastric emptying or even constipation [45]. One reason for this is that there is an increase in the harmone progesterone which slows muscle contractions in the intestines [46]. Another reason is that the intestinal tract becomes squished as the baby grows, causing slow transit of bowel contents through the intestines [47]. Therefore, the chance to have bowel movements in the 10 min examination is very small and their influence on the counts felt by the mothers is litter and ignorable.

Fetal movement can cause distortions over a large area of the abdomen with the intensity of movement and region affected by the movement inherently variable. Therefore, in the current design eight FBG sensors were used to cover a region about 30 cm × 30 cm. It was observed that for some of the fetal movements, all the FBG sensors responded. For other fetal movements, only a few of the FBG sensors responded. This proves the necessity to use an array of FBGs to avoid missing movements, therefore increasing the accuracy of the measurements. FBG-based strain sensors have great multiplex capability—one optical fibre, for example, can have 16 satellite FBG’s, so an array of several optical fibers can be used simultaneously to quickly increase capacity for sensing. Embedding the sensors within the membrane may contribute to improved repeatability and durability and should mitigate user error during data collection due to improper sensor placement.

In this experiment, the volunteers had to lie motionlessly on a sofa while performing the test. This is because movements of the mother could lead to a change of the strain to the optical fibre and therefore could be reflected as distortions to the breathing signals. Similarly, talking, coughing, and sneezing could also contribute to measurement errors. During all the tests there were no cases of coughing and sneezing. In the future work these motion artifacts could be reduced using a reference subtraction method. The reference sensor can be put at a location that will not be affected by the fetal movement, for example, at the side of the wrist, closer to the back. The reference sensor would have a response to the motion artefacts but would not produce any signals due to fetal movements. Therefore, distortions observed by the reference sensor could be subtracted from the signals of the other FBG sensors and motion artifacts could be minimized. Due to the multiplex capability of the FBG based optical fibre sensors [48], the reference FBG can be fabricated at the same optical fibre that is used for fetal movement detection. This simplifies the sensing system and avoids requirement for another channel of the interrogator which can increase the cost. Further, ultrasound imaging continues to be the gold standard method in fetal movement monitoring, so it is important to cross check the sensitivity, accuracy, and the reliability using standard ultrasound equipment, when developing any future FBG sensing device.

With the signal processing algorithms used in the paper, the fetal movements are shown as cluster of spikes and it is difficult to analyses the types of the fetal movements. This is probably because the high-pass filter removed some of the slower signal features of the fetal movements. To be able to categorize the types of the movements, as well as distinguish fetal movements from interferences caused by digestion or abdominal distension, it is necessary to know their individual signal features. These will be one of the future works. Further, fetus heartbeat is an important proxy to the health of the fetus and we will explore in the next steps whether the sensing structure can be used to detect the heartbeat signals simultaneously.

## 5. Conclusions

A successful novel technique for fetal movement monitoring has been investigated and demonstrated. The study suggests that the optical fibre sensors are more sensitive than the mother’s perception of fetal movements. This technique provides many advantages over the conventional more sophisticated and expensive techniques, such as the multiplex capability, the minimal size and weight. Future work will include removal of motion artefacts, validation of the prototype using ultrasound imaging, and the categorization of the type of movements. By addressing these constraints, a wearable belt could be developed for continuous monitoring of fetal movements.
